# Development and stability of Th17 cells in ovarian cancer requires nitric oxide and endogenous NOS2 activity in cancer-associated CD4+ T cells

**DOI:** 10.1186/2051-1426-1-S1-P146

**Published:** 2013-11-07

**Authors:** Natasa Obermajer, Jeffrey L Wong, Robert P Edwards, Kong Chen, Melanie Scott, Shabaana Khader, Jay K Kolls, Kunle Odunsi, Timothy R Billiar, Pawel Kalinski

**Affiliations:** 1 Department of Surgery, University of Pittsburgh, Pittsburgh, PA, USA; 2 Department of Immunology, University of Pittsburgh, Pittsburgh, PA, USA; 3 Department of Infectious Diseases and Microbiology, University of Pittsburgh, Pittsburgh, PA, USA; 4 Magee-Womens Research Institute Ovarian Cancer Center of Excellence, Pittsburgh, PA, USA; 5 Peritoneal/Ovarian Cancer Specialty Care Center, Pittsburgh, PA, USA; 6 University of Pittsburgh Cancer Institute, University of Pittsburgh, Pittsburgh, PA, USA; 7 Department of Pediatrics, University of Pittsburgh School of Medicine, Pittsburgh, PA, USA; 8 Departments of Gynecologic Oncology and Immunology, Roswell Park Cancer Institute, Buffalo, NY, USA

## 

Th17 cells play reciprocal roles in different forms and at different stages of cancer. We report that the presence of Th17 cells in ovarian cancer ascites correlates with local expression of nitric oxide synthase-2 (NOS2). Furthermore, the development of RORγt+IL-23R+IL-17+ Th17 cells from human naive-, memory- or tumor-infiltrating CD4+ T cells critically depends on NO and endogenous NOS2 induced in CD4+ T cells by Th17-inducing cytokines (IL-1β/IL-6/IL-23) or by cancer-associated IL-1β/IL-6/IL-23/NO-producing MDSCs. Inhibition of NOS2 or its downstream cGMP/cGK signaling pathway abolishes de novo induction of Th17 cells. Moreover, even short-term blockade of NOS/cGMP suppresses the IL-17 production by established Th17 cells isolated from ovarian cancer patients, demonstrating the novel key role of NOS/cGMP in Th17 cell physiology and providing for new therapeutic targets to manipulate Th17- and NOS/cGMP-associated immunity in precancerous lesions and advanced cancer.

